# Occupational Sharps and Needlestick Injuries Among Physician Residents at an Academic Medical Center

**DOI:** 10.63564/jha.v14n1p34

**Published:** 2025

**Authors:** Alexei Krainev, Wali Jahangiri, Sofia Villaveces, Hannah Phipps, Victoria Wulsin, Kermit G. Davis, Gordon Lee Gillespie

**Affiliations:** 1 Department of Environmental and Public Health Sciences, University of Cincinnati, Cincinnati, United States; 2 Department of Population Health, University of Cincinnati, Cincinnati, United States; 3 Programming Business Unit, National League for Nursing, District of Columbia, United States

**Keywords:** Needlestick Injuries, Nursing, Physicians, Occupational Injuries, Hospitals, Surveys and Questionnaires

## Abstract

**Objective:**

Occupational sharps and needlestick injuries (SNSI) are a significant and persistent challenge in the U.S. healthcare work environment. With the purpose of better delineating contributing factors for a ubiquitous occupational injury among healthcare workers, we undertook a two-component study of SNSIs among physician residents and nurses at an academic medical center.

**Methods:**

Retrospective injury data among nurses (N=58) and medical residents (N=63) were analyzed. A 35-item cross-sectional survey was used to evaluate the prevalence, non-reporting, and contributing factors among physician residents who sustained a SNSI (N=76).

**Results:**

Physician residents had a rate of injury that was 11.0 SNSIs/100 medical residents/year compared to nurses at 3.2 SNSIs/100 nurses/year; a rate three-fold higher. Physician residents in neurosurgery, otolaryngology, OB/GYN, and general surgery reported the highest rates of injury.

**Conclusions:**

Our results underscore the need for a more comprehensive study to better identify injury drivers specific to the operating room environment.

## 1. Introduction

Occupational sharps and needlestick injuries (SNSI) are a significant and persistent challenge in the U.S. healthcare work environment. A SNSI is defined as any percutaneous, or skin-penetrating, injury to the healthcare worker sustained from a blood-contaminated instrument during patient care. These instruments range from hypodermic needles, stylets, surgical suture needles, and orthopedic pins and rods.^[1]^ Annually, there are approximately 385,000 SNSIs among the U.S. healthcare workforce, with nurses and physicians accounting for the greatest burden of injury.^[1]^ Further, there are 20 known bloodborne pathogens, which have the potential for transmission through percutaneous inoculation from a SNSI, including HIV, hepatitis B, and hepatis C.^[2]^

Employee anxiety, absenteeism, lost productivity, occupational health evaluation, serologic testing and post-exposure HBV/HIV prophylaxis are among some of the direct and indirect costs associated with this ubiquitous occupational injury. The median aggregate cost, including direct and indirect costs, associated with one SNSI is estimated at $747.^[3]^ The total aggregate cost among the U.S. healthcare workforce is estimated to be a minimum of $118 million annually.^[4]^

U.S. Centers for Disease Control and Prevention surveillance data derived from the National Surveillance System for Healthcare Workers (NaSH) from 1995–2003 demonstrate that nurses accounted for most SNSIs (44%) followed by physicians 28%.^[1]^ A total of 23,197 reports of bloodborne pathogen exposures were registered during this time period including SNSIs. Similarly, World Health Organization (WHO) reported trends indicate that nurses and physicians carry the greatest injury burden.^[5]^ According to two multi-hospital SNSI reporting databases, EPINet Sharps Injury Surveillance Research Group (EPINet) and Massachusetts Sharps Injury Surveillance System (MSISS), needlestick injuries have been on the rise over the past decade.^[6]^ Self-report of SNSIs is estimated to be at 23 to 103 needlestick injuries per 1,000 health care workers.^[7]^ In all, SNSIs have a significant impact on healthcare workers.

To better understand SNSIs, a determination of where they occur is necessary. The operating room has been identified as the department with the greatest number of SNSIs.^[6]^ Of note, these types of injuries are of particular relevance to physician residents. As many as 99 of all surgical medical residents are reported to have sustained an SNSI during their medical careers.^[8]^ Moreover, 51% of SNSIs occurring during surgical residency are believed to be unreported.^[8]^

With the purpose of better delineating contributing factors for a ubiquitous occupational injury among healthcare workers, we undertook a two-component study of SNSIs among physician residents and nurses at an academic medical center.

## 2. Methods

The study was divided into 2 components: (1) retrospective phase and (2) cross-sectional survey phase. The retrospective phase reviewed data from an electronic employee injury reporting system from 10/2020 to 11/2021 to evaluate prevalence of institutional OSHA-recordable SNSIs. The cross-sectional survey phase consisted of an anonymous 35-item paper survey distributed to nurses and physician residents.

### 2.1 Retrospective Phase

The retrospective SNSI data consisted of SNSI events among nurses and physician residents that were recorded in an electronic employee health database known as Readyset from 10/2020 to 11/2021 and de-identified to protect employee anonymity. Readyset is an electronic health database through which employees, including nurses and physician residents, report all institutional workplace injuries. The database logs variables including date and time of injury, location of injury, department or division, job title, hire date, needle type, and activity upon occurrence.

Crude injury rates were calculated using Readyset numerator data and denominator data from the institutional human resources department. The following denominator data were obtained from the human resources department: total number of nurse and physician resident hours worked and number of nurses and physician residents at risk. Physician residents were divided into either surgical or non-surgical categories. Non-parametric statistical tests then were performed using the program R.

### 2.2 Cross-Sectional Survey Phase

#### 2.2.1. Instrumentation

An anonymous 35-item 2-page paper survey was generated to assess contributing factors related to SNSI, lifetime injury prevalence, and injury non-reporting among physician residents and nurses. In addition to a comprehensive literature review, a five-member expert panel helped to generate the survey content. This panel consisted of one industrial hygienist, one doctorally prepared occupational health nurse, and three physicians. Members of the panel were experts in the fields of industrial hygiene, ergonomics, occupational health nursing, and medicine and helped with the literature review.

The cross-sectional survey addressed the background of each nurse or physician resident respondent regarding: gender, race, position, level of training, and specialty. The survey then asked whether the respondent experienced a SNSI in the last 12 months, how many, and if they had reported it. Among the examined factors were division of SNSI by type of needle, contributing factors to the injury, shift length during most recent injury, intended task, and if applicable, reasons for non-reporting of the injury. Likewise, needle recapping also was addressed in the survey as it is a known risk factor for SNSI.^[9]^

#### 2.2.2. Procedures

Surveys were solicited through the Graduate Medical Education Office at monthly physician resident lunches and annual physician resident in-service exams. The respondents placed the completed paper surveys into opaque envelopes and then deposited them into locked boxes placed in each department’s mailroom. Respondents were asked to submit the survey regardless of survey completion.

### 2.3. Data Analysis

Physician residents were divided by surgical and non-surgical specialty. OSHA-recordable SNSI injury data were queried on ReadySet and then imported into Microsoft Excel. Individual employee identifiers were removed, Crude rates were calculated by obtaining human resources department data for the corresponding 2020–21 fiscal year. Crude rates were calculated for SNSIs among nurses and physician residents.

### 2.4. Ethical Considerations

Review of de-identified OSHA-recordable employee injury records and solicitation of an anonymous paper survey were conducted following approval by the Institutional Review Board under University of Cincinnati IRB #2022-0364.

## 3. Results

### 3.1 Retrospective Phase

Nurses and physician residents made up 33% and 54% of all 138 reported institutional SNSIs, respectively. The crude SNSI rate for nurses was 3.2 SNSIs/100 nurses/year and for physician residents was 11 SNSIs/100 physician residents/year (see Table 1). When adjusted per hours worked, nurses had a rate of 2 SNSIs/100,000 hours whereas residents had a rate of 4 SNSIs/100,000 hours.

**Figure 1.**
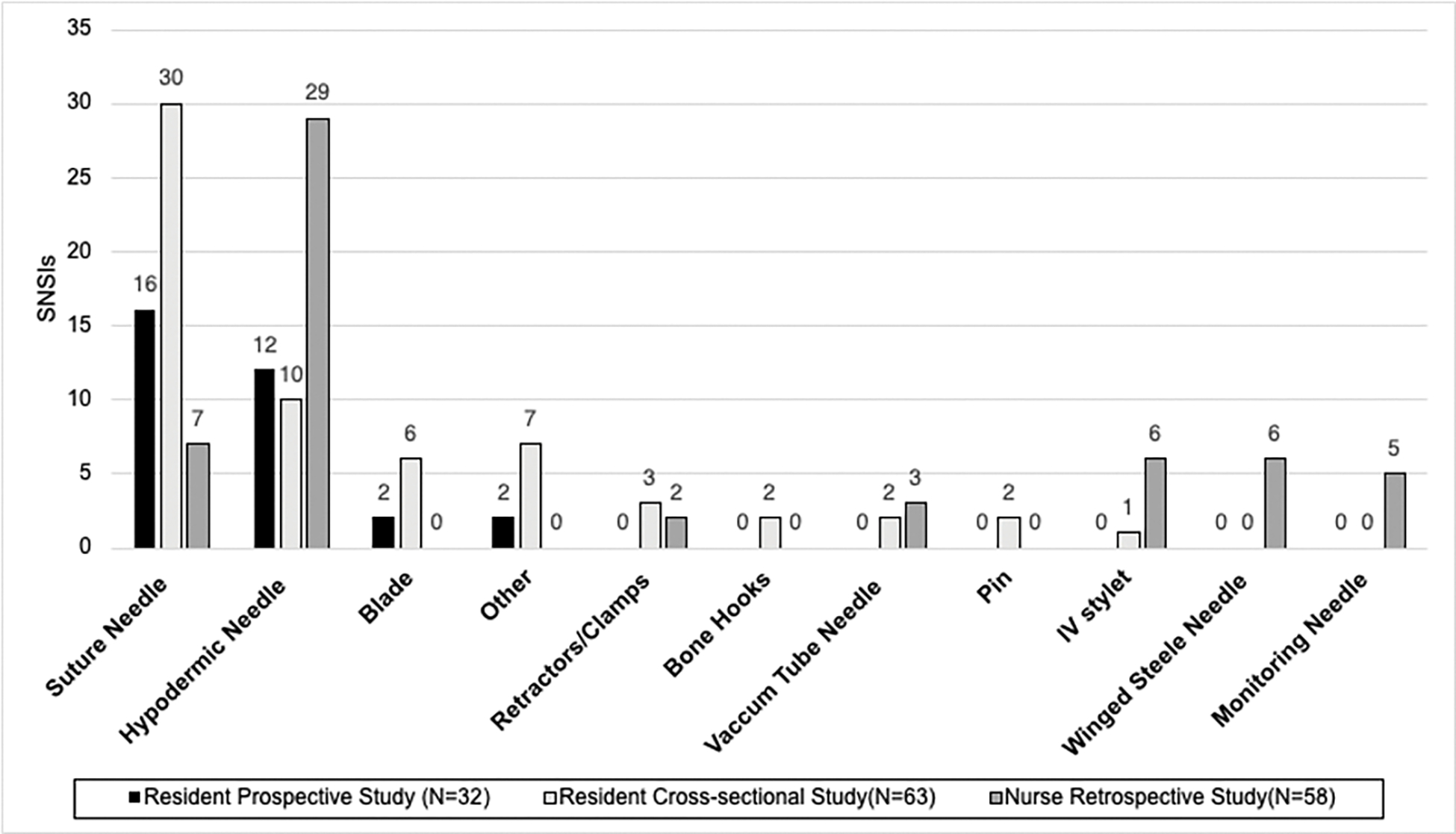
Instrument use and SNSI among physician residents and nurses. The greatest number of SNSIs among physician residents occurred due to suture needles. Most SNSIs among nurses were associated with use of hypodermic needles followed by suture needles.

**Figure 2.**
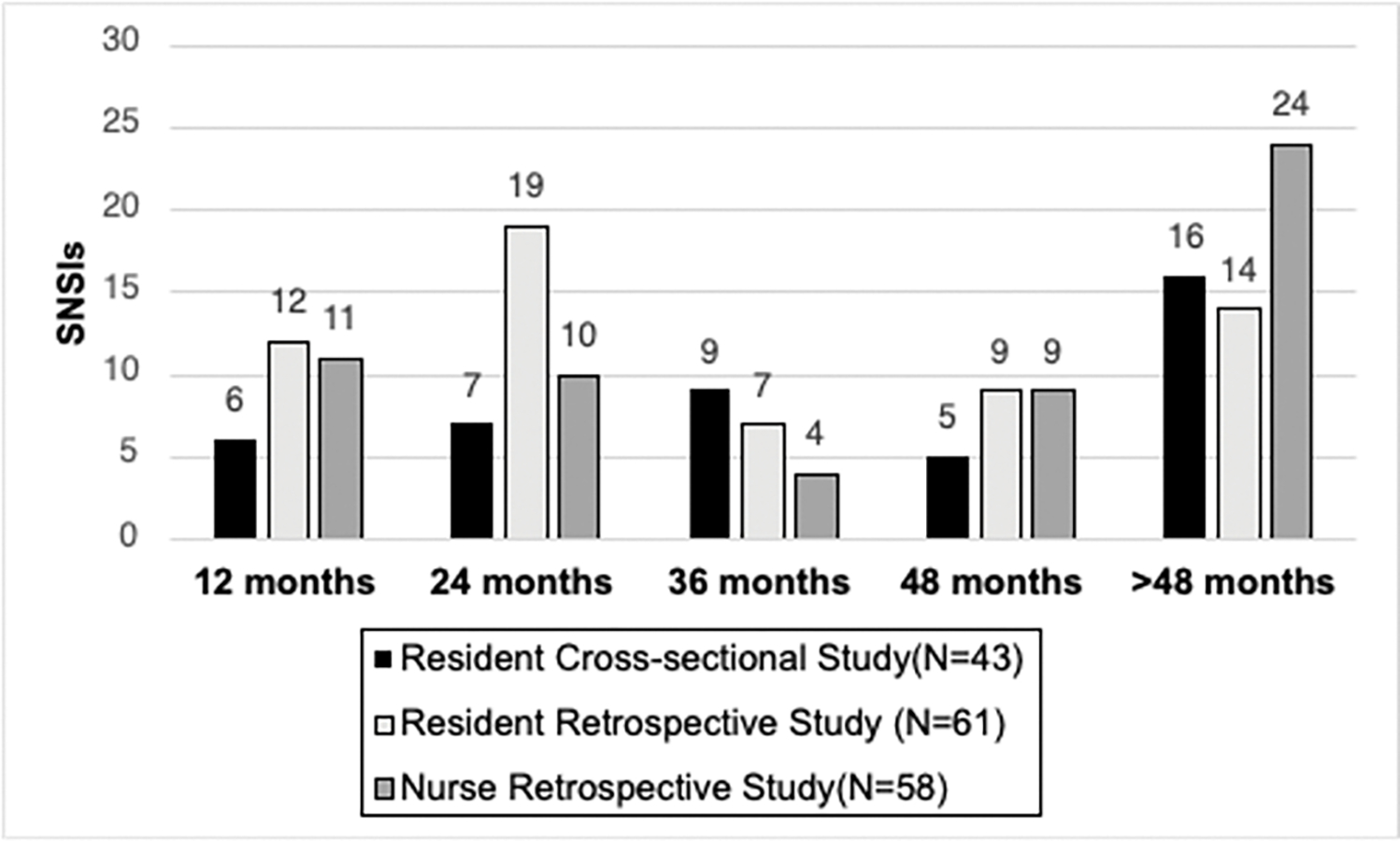
SNSIs among physician residents and nurses compared by date of employment initiation. SNSIs among physician residents peaked at 24 months and 48+ months following the date of employment. Among nurses, SNSIs peaked at 48+ months.

Among physician residents, those in a surgical specialty had a significantly higher number of recorded SNSIs. A surgical physician resident specialty was defined to include: general surgery, orthopedics, otolaryngology (ENT), obstetrics and gynecology (OB/GYN), podiatry, ophthalmology, urology or neurosurgery. Physician residents were most likely to be injured by a suture needle or hypodermic needle during work (see Figure 1). Suture needles made up 47% of all recorded injuries in the retrospective study. Among residents in a surgical specialty, those in general surgery, otolaryngology (ENT), OB/GYN and neurosurgery carried the greatest burden of SNSI. Physician residents’ SNSIs peaked at 24 months following the start of employment and then again at greater than 48 months after the start of employment (see Figure 2). Otolaryngology or ENT recorded the highest rate of SNSI at 58 SNSIs/100 physician residents/year when rate of injury was evaluated for each individual specialty (see Table 2). Neurosurgery, OB/GYN, and general surgery likewise had rates significantly higher than the aggregate resident rate (0.11).

### 3.2 Cross-sectional Phase

**Figure 4.**
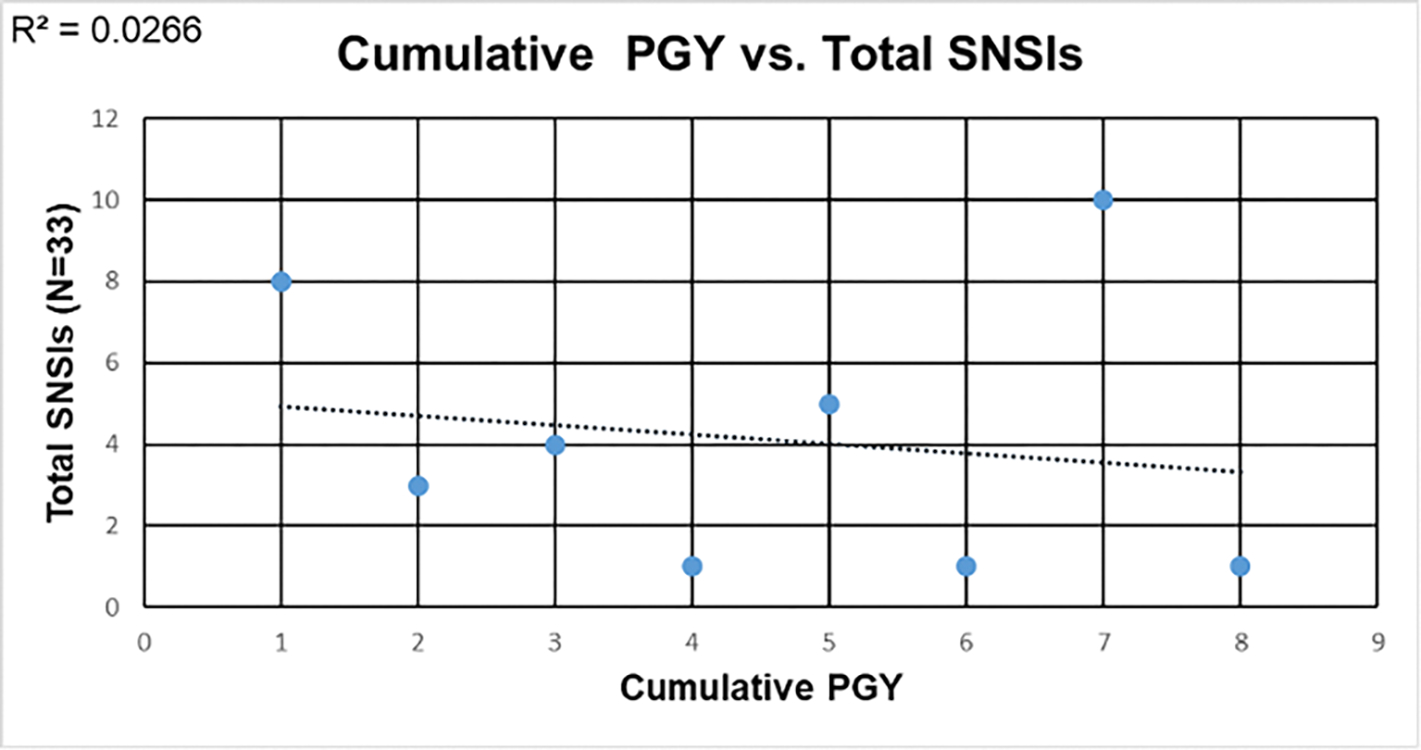

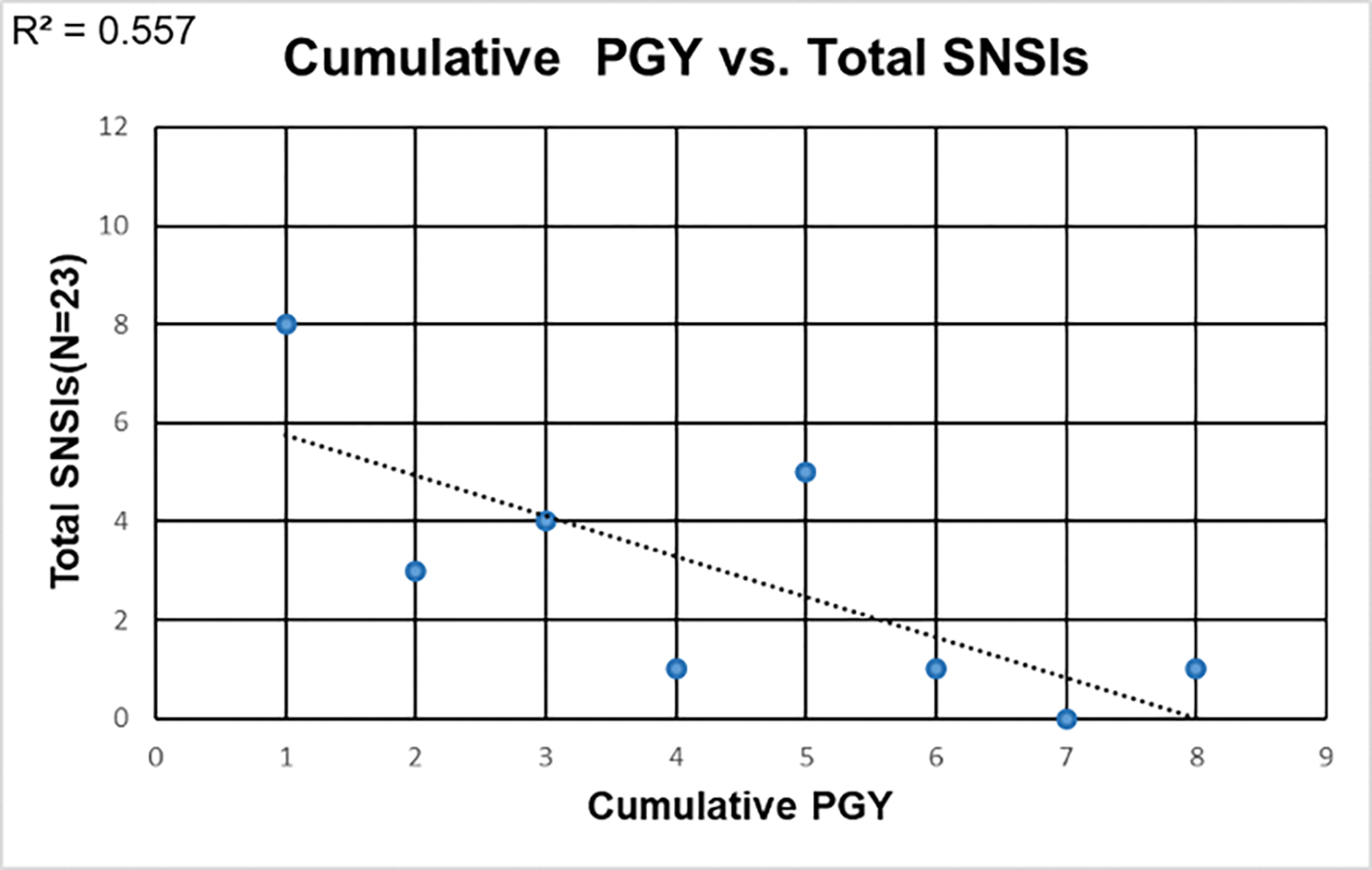
Cumulative postgraduate training year and total recorded SNSIs among physician residents. (4a) There is a weak negative association between cumulative postgraduate year and number of SNSIs (R2=0.027). (4b) When a single physician resident with 10 SNSIs is removed, it increases (R2= 0.48).

In the cross-sectional survey (N=76), 96.1% (n=73) of physician resident respondents reported a history of a SNSI during their career (see Table 3). Among those with a prior history of injury, general surgery physician residents accounted for the highest number of SNSIs, followed by anesthesia and internal medicine. When plotting cumulative postgraduate resident training as the independent variable and SNSIs as the dependent variable; there is a weak negative association (see Figures 4a & 4b). As cumulative postgraduate training increases, the number of recorded SNSIs decreases. Among physician residents, 38% reported having recapped a hollow-bore hypodermic needle during the preceding 12 months. Among the physician residents with a prior history of a SNSI, most occurred while working a 12-hour shift. Some (16%) of the physician resident respondents also indicated they were fearful that reporting the SNSI would reflect poorly on their perceived performance. Finally, among physician residents, 41% of respondents who had a previous SNSI indicated that they had not reported it.

## 4. Discussion

SNSIs among physician residents in the operating room constitute a persistent occupational health challenge. Several important findings were noted from our retrospective and cross-sectional studies. First, physician residents carry a greater burden of SNSIs compared to their nursing counterparts. Physician residents had a three-fold higher rate of SNSI compared to nurses. Likewise, when compared to NaSH CDC surveillance data, the physician resident rate is more than threefold higher than the national average reported for physicians (2 SNSIs/100 physicians per year).^[1]^ In contrast, the rate of SNSI among nurses was found to be lower than the reported NaSH rate of 9 SNSIs/100 nurses per year.

Several factors were identified that contributed to the observed increased rate of SNSIs among physician residents. A significant factor was, in part, the result of them being in training. Procedural tasks are delegated to physician residents because they seek to undertake more procedures (i.e., central line placement, suturing, and peripheral intravenous line placement) in order to become more competent in the procedural tasks enumerated by their training programs. Physician residents’ dedication to their learning is noteworthy. However, it is possible that the tasks are delegated to physician residents without adequate oversight to assure their safety while performing new tasks. As postgraduate training experience increased among the physician residents, there was a corresponding linear decrease in the number of SNSIs. This relationship reflects that over time the physician residents are becoming more competent and have learned to safely handle sharps and needles. It is important to note that enhanced prevention efforts are needed to prevent these early career SNSIs. Closer and direct observation of physician residents by attending physicians or even potentially nurses and surgical assistants as sharps and needles are being used may be warranted to prevent inadvertent SNSIs.

A second contributing factor to SNSIs among physician residents was their subspecialty. Surgical subspecialties carried the greater burden of SNSI among physician residents. Surgical residents, especially those in ENT, OB/GYN, neurosurgery, and general surgery had a significantly higher rate of injury than the overall physician resident crude rate. Surgical residents spend a larger portion of their working time in the operating room where they are more likely to come in contact with disposable suture needles during surgical procedures. Redesigning the procedures for management of sharps and needles when physician residents (and others in the operating room environment) are done with them could be considered. For example, a cork be added to suture trays and as a procedure such as suturing is nearly complete, the surgical assistant could remind the person conducting the suture placement to drive the suture needle into the cord to minimize the risk of a SNSI. The cork becomes a much more visible identifier of a sharp or needle location. Hypodermic needle recapping is likewise a persistent problem. To prevent a recapping injury, a clean conical tube can be placed on instrument trays such as a needle or scalpel can be placed into the tube until it is ready for disposal.^[10]^ Of utmost importance is that attending physicians model and reiterate safety practices as they personally handle sharps and needles.

Of concern, the rate of non-reporting of SNSIs among physician residents is 41 SNSIs/100 physician residents per year, higher than the healthcare worker average (which includes nurses, medical assistants, physicians, and physician residents) reported in the literature.^[7]^ Furthermore, in the cross-sectional survey phase, 16% of physician resident respondents were fearful that reporting a SNSI would reflect poorly on their performance. All in all, the observed trends are consistent with the persistent nature of these occupational injuries among the physician resident worker population and support the need to address any misconceptions about reporting on a systems level. To promote a culture of reporting, attending physicians can discuss their own experiences with suffering from a SNSI during the course of their career, describe the importance of reporting injuries as they occur, and assuring physician residents that they will not be penalized for experiencing or reporting a SNSI.

### 4.1 Limitations

Despite the cross-sectional survey being anonymous, one limitation is survey bias; specifically, response bias. This bias is related to respondent hesitancy to answer truthfully about non-reporting of SNSIs and fear of perceived retaliation given that the survey is conducted in an occupational setting. Furthermore, the rates obtained from the retrospective phase rely on passive surveillance through employee self-report of injury. As is the case with the majority of reportable infectious diseases, passive surveillance is often associated with underreporting.^[11]^ Underreporting also can result from the lack of awareness of reporting procedures and negative worker attitudes towards injury reporting.^[11]^

## 5. Conclusions

Our work supports the need to implement interventions that focus on the use of different sharps instruments on medical/surgical floors versus the operating room, as sharps hazards present in the operating room warrant correspondingly different interventions and analyses. The operating room environment is complex and involves an interplay of multiple workers handling sharps instruments including: physician residents, attending surgeons, and surgical assistants. Patient positioning, operator posture, and instrument hand off techniques during surgeries coalesce to create a complex occupational environment that creates different drivers of SNSI than those present on a medical/surgical floor. An ergonomic analysis of SNSIs in the operating room could delineate drivers of injury among surgical physician residents.

## Figures and Tables

**Figure 3.**
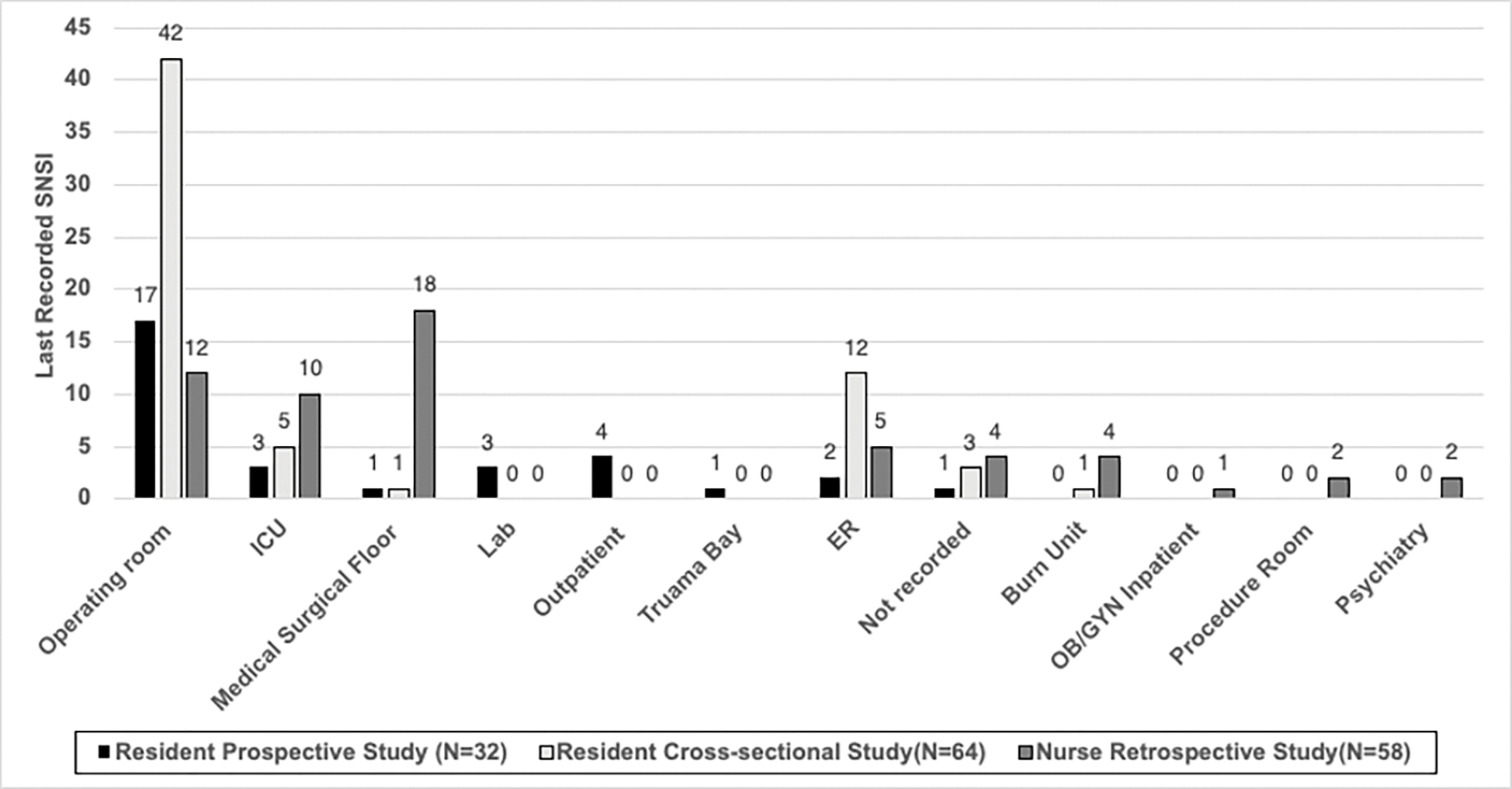
Most SNSIs among resident physicians occurred in the operxxating room according to the cross-sectional survey followed by the outpatient setting. Among nurses, most SNSIs occurred in the medical surgical ward (31%) followed by the operating room (21%).

**Table 1.** Crude institutional SNSI rates for nurses and resident physicians.

Variable	Nurses	Physician Residents

Total number	1801	579
Total hours worked	2 943 784	1 437 081
Recorded SNSI events	58	64
Number SNSI per 100,000 hours worked	2	4
Number SNSI per 100 persons	3.2	11

**Table 2.** Retrospective phase: Sharp/needlestick injury (SNSI) rate by physician resident specialty.

Specialty	Rate	95% CI	p-value

Aggregate resident rate	0.11	-	-
Otolaryngology	0.58	0.330–0.800	<0.0001
Neurosurgery	0.38	0.140–0.680	0.01
OB/GYN	0.30	0.160–0.470	0.002
Ophthalmology	0.21	0.050–0.510	0.20
General Surgery	0.20	0.110–0.310	0.02
Orthopedics	0.20	0.08–0.390	0.13
Podiatry	0.14	0.004–0.580	0.56
Emergency Medicine	0.10	0.040–0.210	1.00
Urology	0.10	0.002–0.450	1.00
Dermatology	0.09	0.002–0.410	1.00
Pathology	0.07	0.002–0.340	1.00
Radiology	0.03	0.001–0.140	0.18
Anesthesia	0.03	0.001–0.140	0.12
Internal Medicine	0.004	0.0001–0.030	<0.0001

**Table 3.** Descriptive findings from cross-sectional survey phase with physician residents (n=76).

Variable	Frequency	Percentage

Biological sex*		
Male	21	61.8
Female	13	38.2
Current postgraduate year training level**		
1	6	14
2	7	16.3
3	9	20.9
4	5	11.6
5	4	9.3
6	7	16.3
7	4	9.3
8	1	2.3
Specialty***		
General surgery	22	46.8
Internal medicine	8	17
Anesthesia	5	10.6
Neurology	3	6.4
Pathology	3	6.4
ENT	2	4.3
Psychiatry	2	4.3
Radiation oncology	1	2.1
Radiology	1	2.1
Total career sharp/needlestick injury	73	96.1
Instrument/device (n=32)		
Suture needle	16	50
Hollow-bore needle	12	37.5
Blade	2	6.3
Glass shard	1	3.1
Not recorded	1	3.1

* missing data for 42 participants

** missing data for 33 participants

*** missing data for 29 participants
